# The Role of Large Language Models (LLMs) in Providing Triage for Maxillofacial Trauma Cases: A Preliminary Study

**DOI:** 10.3390/diagnostics14080839

**Published:** 2024-04-18

**Authors:** Andrea Frosolini, Lisa Catarzi, Simone Benedetti, Linda Latini, Glauco Chisci, Leonardo Franz, Paolo Gennaro, Guido Gabriele

**Affiliations:** 1 Maxillofacial Surgery Unit, Department of Medical Biotechnologies, University of Siena, 53100 Siena, Italy; lisa.catarzi@student.unisi.it (L.C.); simone.benedetti@student.unisi.it (S.B.); latilinda94@gmail.com (L.L.); glauco.chisci@gmail.com (G.C.); paolo.gennaro@unisi.it (P.G.); guido.gabriele@unisi.it (G.G.); 2 Phoniatris and Audiology Unit, Department of Neuroscience DNS, University of Padova, 35122 Treviso, Italy; leonardo.franz@unipd.it; 3 Artificial Intelligence in Medicine and Innovation in Clinical Research and Methodology (PhD Program), Department of Clinical and Experimental Sciences, University of Brescia, 25121 Brescia, Italy

**Keywords:** Large Language Models (LLM), GEMINI, ChatGPT, maxillofacial, trauma, triage, AIPI, QAMAI, maxillofacial surgery

## Abstract

Background: In the evolving field of maxillofacial surgery, integrating advanced technologies like Large Language Models (LLMs) into medical practices, especially for trauma triage, presents a promising yet largely unexplored potential. This study aimed to evaluate the feasibility of using LLMs for triaging complex maxillofacial trauma cases by comparing their performance against the expertise of a tertiary referral center. Methods: Utilizing a comprehensive review of patient records in a tertiary referral center over a year-long period, standardized prompts detailing patient demographics, injury characteristics, and medical histories were created. These prompts were used to assess the triage suggestions of ChatGPT 4.0 and Google GEMINI against the center’s recommendations, supplemented by evaluating the AI’s performance using the QAMAI and AIPI questionnaires. Results: The results in 10 cases of major maxillofacial trauma indicated moderate agreement rates between LLM recommendations and the referral center, with some variances in the suggestion of appropriate examinations (70% ChatGPT and 50% GEMINI) and treatment plans (60% ChatGPT and 45% GEMINI). Notably, the study found no statistically significant differences in several areas of the questionnaires, except in the diagnosis accuracy (GEMINI: 3.30, ChatGPT: 2.30; *p* = 0.032) and relevance of the recommendations (GEMINI: 2.90, ChatGPT: 3.50; *p* = 0.021). A Spearman correlation analysis highlighted significant correlations within the two questionnaires, specifically between the QAMAI total score and AIPI treatment scores (rho = 0.767, *p* = 0.010). Conclusions: This exploratory investigation underscores the potential of LLMs in enhancing clinical decision making for maxillofacial trauma cases, indicating a need for further research to refine their application in healthcare settings.

## 1. Introduction

In the dynamic landscape of modern maxillofacial surgery, the intersection of advanced technology and daily medical practice is continuously evolving [1,2]. This is particularly evident in traumatology, a field where the complexity and urgency of injuries demands not only a multidisciplinary management but also innovative solutions for efficient triage and treatment planning. Maxillofacial trauma presents a unique set of challenges in medical triage due to the anatomical complexity and the critical functional and aesthetic roles of the facial region [3]. The variability in injury mechanisms—ranging from traffic collision to interpersonal violence—leads to the incidence of multiple fractures and trauma-related vascular and parenchymal damage potentially involving all body organs [4]. These complex cases further complicate the diagnosis, prioritization, and treatment planning processes [5]. Traditional triage systems may struggle to rapidly assimilate and analyze the diverse and complex data associated with such injuries, potentially delaying critical interventions [6].

In the last few years, the effectiveness of telemedicine in enhancing the decision-making process for the appropriate care setting of facial trauma patients, thereby potentially minimizing unnecessary specialized facility transfers have led to increased application of such technology [7]. Moving forward from this actual practice and imagining the next future developments, we hypothesized that the integration of Large Language Models (LLMs) in the maxillofacial traumatology triage process may be a promising yet uncharted territory. LLMs are advanced AI-driven systems designed to understand, interpret, and generate human language. Built on extensive neural network architectures, such as transformers, these models are trained on vast datasets of text to learn language patterns, context, and semantics. Their ability to understand and generate coherent, contextually relevant responses makes them valuable in various applications, extending to fields like natural language processing, content creation, customer service, and increasingly, healthcare [8]. LLMs are currently explored as valuable tools in surgical planning and various other medical fields, including clinical decision making, biomedical research, and healthcare education, as suggested by the recent literature [9,10]. In the context of emergency triage, an original study aimed to assess the efficacy of ChatGPT and Google Bard, compared to medical students, in conducting Simple Triage And Rapid Treatment (START triage) in mass casualty incidents. The authors found that Google Bard exhibited a notably higher accuracy rate of 60%, surpassing ChatGPT’s 26.67% accuracy, and closely matching the 64.3% accuracy rate of medical students [11]. Integrating LLMs into the triage and treatment planning for maxillofacial trauma can offer significant advantages: (i) as LLMs can understand and process complex medical histories and incident reports, a rapid preliminary assessment of the patient’s condition—including the identification of critical details that might affect triage decisions, such as the mechanism of injury, the presence of comorbidities, and initial signs and symptoms—can be performed; (ii) by analyzing vast datasets, LLMs can recognize patterns and correlations that might not be immediately apparent to human clinicians, thus improving the triage both at individual level—enabling a personalized approach to triage and treatment—and at a population level—ameliorating the triage system itself and implementing preventive interventions; (iii) decision support can be provided by suggesting triage and treatment pathways grounded in the latest medical research and guidelines (i.e., quickly sift through the medical literature to find relevant studies, guidelines, and case reports tailored to the specificity of each case); (iv) better communication and coordination can be facilitated among the various specialists involved in complex cases requiring multidisciplinary (e.g., generating comprehensive summaries and recommendations based on the patient’s data, ensuring that all team members have access to the same information); (v) LLMs can be used to create detailed simulations of maxillofacial trauma cases for training purposes; (vi) deploying LLM-based systems can make expert-level triage recommendations more accessible, especially in under-resourced settings where specialist availability is limited.

While LLMs offer promising opportunities, there are several drawbacks and challenges that must be considered: (i) processing sensitive patient information raises significant concerns about data privacy and security, necessitating robust data protection measures; therefore, LLMs use should be subject to rigorous regulatory scrutiny to ensure their safety and efficacy; (ii) large datasets may contain inherent biases that can be perpetuated and even amplified (e.g., if the data are predominantly from a certain demographic, the model might perform less accurately for patients outside of that geographical area, thus exacerbating health inequalities); (iii) healthcare providers might become overly reliant on LLMs for decision making, potentially leading to complacency if the AI suggestions are accepted without sufficient critical evaluation; (iv) as the models’ internal workings are often opaque—resembling a “black box”—the recommendations made can be difficult to interpret, raising questions about accountability in cases of misdiagnosis as LLMs—despite their advanced capabilities—are not infallible and can generate incorrect or irrelevant recommendations; (v) medical knowledge and guidelines can change rapidly, and LLMs might not immediately reflect the most current information unless they are continually updated; (vi) developing, training, and implementing LLM-based systems can be costly and resource-intensive (there is a need for substantial computational resources, as well as ongoing maintenance and updates to the models), thus posing significant barriers for smaller healthcare facilities and for low-resource healthcare systems.

To the best of our knowledge, no studies aiming to explore the use of LLMs in maxillofacial trauma triage have been published. Given this background, our aim was to conduct an exploratory investigation to preliminarily assess the feasibility of an LLM-based triage modality in supporting clinical decision making for complex maxillofacial trauma cases. As a primary aim, we focused on the level of agreement between the proposed LLM-based triage process and tertiary referral center real-life experience. As a secondary aim, we evaluated the AI’s performance with existing validated questionnaires to rank their answer within the scope of the study.

## 2. Materials and Methods

### 2.1. Patients and Triage System

We comprehensively reviewed the medical chart of patients treated from 1 January 2023 to 31 December 2023 at a tertiary referral center for head and neck fractures (Maxillofacial Surgery Unit, Department of Medical Biotechnologies, University of Siena, Italy). Inclusion criteria were as follows: (i) major traumas defined as significant injury or injuries that have potential to be life-threatening or life-changing sustained from either high-energy mechanisms or low-energy mechanisms in those rendered vulnerable by extremes of age/comorbidities [12]; (ii) complete medical and surgical reports and follow-up. The medical and surgical data of included patients were retrieved and prompts were generated in order to ask ChatGPT 4.0 (OpenAI, San Francisco, CA, USA) and Google GEMINI (Google, Washington, DC, USA) to propose a specific triage process and surgical management.

### 2.2. Prompt Design

Standardized case-based prompts were formulated, encompassing detailed patient demographics, specific maxillofacial injury characteristics, medical history, and comorbidities. This approach aimed to simulate the clinical complexity encountered in maxillofacial trauma triage. Uniformity in prompts was maintained across both LLMs, ensuring comparability (see example in Supplementary Materials). The prompts’ clinical relevance was validated by maxillofacial surgery experts to accurately reflect the informational needs for triage and management decisions. Each scenario was input into the AI interfaces sequentially following a strict protocol on 29 February 2024. To avoid any bias and maintain consistency, each case was entered using a new instance of the chat interface, with internet cache data cleared beforehand. A carefully designed standard input phrase was used across all cases: “You are a maxillofacial surgeon receiving the following patient’s documentation: provide the most appropriate triage process based on the presented information”. This was the initial prompt. Furthermore, a second prompt requested the AI to “Please provide references to support your triage choice”. The evaluation of the AI’s suggestions was conducted by comparing its recommended specialist consultations and surgical interventions with those of the tertiary referral center.

### 2.3. LLMs Answer Evaluation: QAMAI and AIPI

An expert surgeon panel of reviewers was composed including six maxillofacial surgeons, each having more than 5 years of specialist experience. The responses of the two LLMs and the ones of the control group were evaluated using the QAMAI tool, an instrument with 6 items, consisting of Likert scales that range from 1 (strongly disagree) to 5 (strongly agree), which assess the accuracy, clarity, relevance, the completeness, and the quality of the references and usefulness, as previously reported [13]. The total score can range from 5 to 30. The Artificial Intelligence Performance Instrument (AIPI) was also employed. This tool consists of nine items, divided into four sub-scores that evaluate the AI’s performance in the areas of considering patient features, suggesting a differential diagnosis, proposing additional examinations, and suggesting a treatment plan. The final score ranged from 0 to 20.

### 2.4. Statistical Analysis

A comparative analysis of ChatGPT and GEMINI responses was conducted using the paired *t*-test to highlight any possible performance discrepancies. A Spearman analysis was conducted to explore the correlation between AIPI and QAMAI questionnaires. A *p* value < 0.05 was considered statistically significant. The statistical software used was Jamovi 2.3 (The Jamovi Project 2022, Sydney, Australia).

## 3. Results

The analysis of the surgical database at our institution resulted in 157 maxillofacial fractures being treated during the study period. Of these, 94 fractures were excluded as not fulfilled criteria of major trauma and 53 were excluded as not having a complete report available, leading to the final inclusion of 10 consecutive cases, as outlined in Table 1. The cohort comprised three females and seven males with a mean age of 38.9 years (SD 16.68 years). The nature of injuries varied significantly, with incidents ranging from car accidents to domestic falls and physical aggression. Notably, the types of maxillofacial fractures encountered were diverse, including injuries to the orbital floor, nasal, maxillary, mandibular, and zygomatic bones, among others.

The ME section of Table 2 outlines the specialist consultations recommended by each entity. ChatGPT’s recommendations varied, including referrals to ophthalmologists, neurologists, orthopedics, infectious disease specialists, and dentists, depending on the case specifics.

GEMINI, in some instances, did not provide specific recommendations (marked as NA—not applicable), while the tertiary referral center’s recommendations included a mix of the specialists listed above. ChatGPT showed a 70% match rate with the tertiary referral center, suggesting a moderate level of agreement. GEMINI’s recommendations matched 50% of the time with the gold standard, indicating a slight lower level of concordance. The treatment section in Table 2 details the therapeutic approaches suggested, including various procedures like open reduction internal fixation (ORIF), orbital floor repair, antibiotics, and post-operative interventions like rehabilitation or intensive care. ChatGPT demonstrated a 60% match rate with the tertiary referral center, indicating a relatively higher consistency in treatment recommendations. GEMINI matched the gold standard in 45% of the cases, showing a fair level of agreement but still behind ChatGPT.

The comparative analysis of AIPI and QAMAI scores for GEMINI and ChatGPT, as comprehensively detailed in the results in Table 3, showed notable variances in performance across a spectrum of items. GEMINI generally performed slightly better than ChatGPT according to AIPI questionnaire evaluations with a statistically significant higher score in diagnosis (GEMINI: 3.30, ChatGPT: 2.30; *p* = 0.032). However, in other areas like additional examinations and treatment plans, differences were not statistically significant, and a higher total score (GEMINI: 9.50, ChatGPT: 7.60; *p* = 0.052) approaching but not reaching statistical significance was retrieved. According to the QAMAI questionnaire, ChatGPT scored slightly higher across most metrics but showed a statistically significant difference only in the relevance item (GEMINI: 2.90, ChatGPT: 3.50; *p* = 0.021). Although ChatGPT overall performance was better in terms of total QAMAI, this difference was not statistically significant (GEMINI: 18.40, ChatGPT: 18.85; *p* = 0.765).

**Figure 1:**
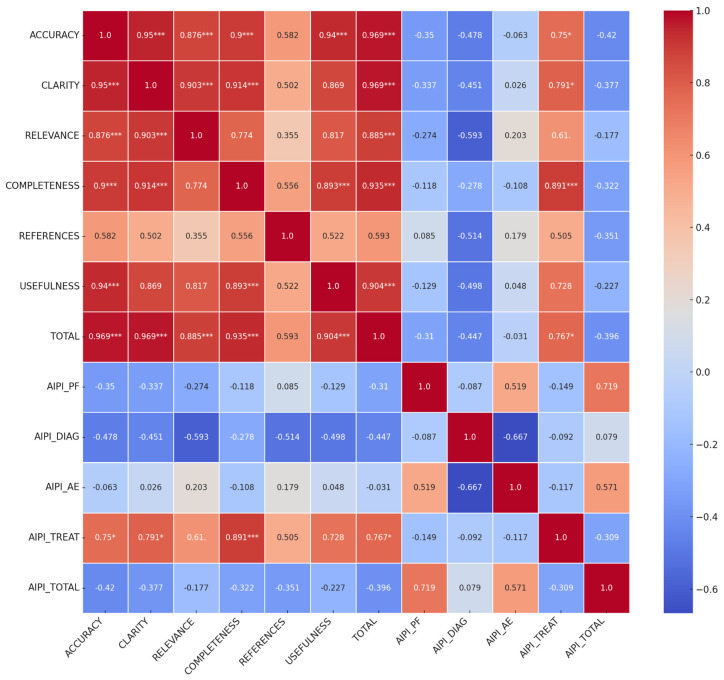
Spearman correlation matrix of ChatGPT’s QAMAI and AIPI scores: the heatmap visualizes the Spearman correlation matrix for various QAMAI scores and AIPI scores related to ChatGPT’s performance. Each cell in the heatmap represents the Spearman correlation coefficient between two variables, with the color intensity and the value indicating the strength and direction of the correlation. Positive correlations are indicated by warmer colors (red), whereas negative correlations are shown with cooler colors (blue). Asterisks indicates significant values: * *p* < 0.05, *** *p* < 0.001.

The Spearman correlation analysis between the QAMAI and AIPI scores revealed significant intra and intercorrelations, as shown in Figure 1. Strong positive correlations were observed within the QAMAI scores themselves. Specifically, the correlation between accuracy and clarity was notably high (Spearman’s rho = 0.950, *p* < 0.001), as was the correlation between accuracy and relevance (rho = 0.876, *p* < 0.001), and between accuracy and completeness (rho = 0.900, *p* < 0.001). A significant positive correlation emerged between the QAMAI total score and AIPI treatment scores (rho = 0.767, *p* = 0.010). Other AIPI dimensions such as patient feature consideration, diagnosis, and additional examinations did not show significant correlations with QAMAI scores. Additionally, a negative correlation was observed between AIPI diagnosis and additional examinations (rho = −0.667, *p* = 0.035).

## 4. Discussion

This study evaluated the performance of ChatGPT and GEMINI in the trauma triage setting, exploring its potential as an aid in the clinical decision making of maxillofacial trauma cases, and ranking the AI’s performance with existing validated questionnaires. Previous research has delved into the application of LLMs within Emergency Triage, with a particular emphasis on aspects such as triage scores, hospitalization rates, and estimations of critical illness [11,14,15,16]. However, according to our knowledge, this is the first study in the literature to investigate both the diagnostic accuracy and appropriateness of triage recommendations of LLMs in response to maxillofacial trauma cases. Moreover, given the recent release of GEMINI, few studies that compare these advanced LLMs have been conducted [17,18]. Our findings confirmed that LLMs hold promise in trauma triage, but improvements are necessary for reliable implementation in clinical practice. During the last year, the application of LLMs in healthcare has dramatically increased [19,20,21,22]. The deployment of these models raises some concerns regarding potential biases in algorithmic decision making, adherence to medical regulatory frameworks, risk of hallucination, and privacy issues [23,24,25,26]. As a result, an empirical validation of their outputs still remains mandatory at this time [27]. The integration of LLMs into established healthcare infrastructures and their reliance on comprehensive high-quality datasets are non-trivial challenges that necessitate prompt and careful consideration [2]. Inappropriate or inaccurate advice remains the largest challenge to integrating a patient-interactive AI triage tool. Among the greatest concerns include a potential delay in patients seeking urgently or emergently needed medical attention following trauma. Legal liability from errors in judgment or delays in care attributed to an AI triage tool may dissuade surgeons from integrating such technology into their practices [28].

Based on our preliminary data, LLMs seem to be at potential risk of underperforming the comprehensive evaluation of complex trauma cases. These concerns, however, are not unique to an AI interface but are similar to concerns present when training new or inexperienced clinical staff that may be triaging patients [29]. In this pilot study, ChatGPT exhibited a slightly higher match rate with the referral center both in specialist consultations and treatment recommendations compared to GEMINI. Regarding multidisciplinary evaluation, ChatGPT showed a 70% match rate with the tertiary referral center. Previous studies suggest that chatbots potentially decrease unnecessary clinical appointments and release valuable resources for those in greater need [30]. However, in our study, LLMs in some cases proposed more additional specialist visits than recommended by the gold standard. At this stage, it is unclear if the AI can provide a more holistic view of patients’ conditions or may be prone to propose unnecessary visits potentially delaying appropriate diagnosis and treatment. It is essential to further explore the clinical relevance and appropriateness of these additional recommendations, in terms of the time spent on and overall cost of an appropriate diagnosis. The negative correlation observed between AIPI diagnosis and additional examinations implies that higher accuracy in diagnosis recommendations by ChatGPT may result in less emphasis on suggesting additional examinations (see Figure 1). While this may indicate a potential limitation of ChatGPT in considering the need for further diagnostic testing, it also highlights the importance of integrating clinical judgment and contextual knowledge alongside AI-generated recommendations to ensure comprehensive patient care. In terms of treatment recommendations, ChatGPT demonstrated a relatively higher consistency with the referral center (60% match rate) compared to GEMINI (50% match rate). This indicates that ChatGPT’s suggestions were more in line with established clinical practices, potentially leading to better patient outcomes. The observed variance in recommendations between ChatGPT and Google GEMINI within our study offers insights into the capabilities of different LLMs, underscoring the importance of the underlying architecture and training datasets in shaping the models’ output. Therefore, our comparison highlights the necessity for interdisciplinary oversight where AI recommendations are evaluated and contextualized by human experts. As delineated in the results in Table 3, GEMINI provided a superiority over ChatGPT in terms of AIPI score and, conversely, underperformed in the QAMAI score. These differences were statistically significant for the “diagnosis” item of the AIPI questionnaire (GEMINI: 3.30, ChatGPT: 2.30; *p* = 0.032) and the “relevance” item of the QAMAI questionnaire (GEMINI: 2.90, ChatGPT: 3.50; *p* = 0.021). These data apparently contrast with the matching percentage of LLMs against tertiary referral centers. Nonetheless, most scores, and specifically the total scores of AIPI and QAMAI, were not statistically different, as specified in Table 3. Additional research is required to elucidate the relationship between the outcomes provided by LLMs and the metrics of evaluation tools like AIPI and QAMAI. Our study examining the connections between two distinct questionnaires revealed multiple correlations within the QAMAI constructs, as well as a significant correlation between the overall QAMAI score and AIPI treatment scores (rho = 0.767, *p* = 0.010), as illustrated in Figure 1. These findings initially support the construct validity of both questionnaires. However, more detailed investigations are necessary to determine threshold values and establish precise guidelines and applications for each questionnaire across various settings. Based on our results, we can assert that the LLMs models effectively recognized all patients who needed surgery. Indeed, in all cases where the gold standard indicated surgery (ORIF), both GEMINI and ChatGPT proposed surgery as the treatment. However, they can struggle with accurately specifying proper surgical treatment details. In particular, in one case, GEMINI inadequately proposed intermaxillary fixation and missed the necessity of teeth splinting in another case, resulting in less appropriateness than ChatGPT in treatment proposals.

Overall, the findings of this study underscore the potential of LLMs as a valuable tool in clinical decision-making processes, particularly in providing accurate and relevant treatment recommendations. However, akin to Patel’s findings, integrating LLMs into clinical settings presents numerous hurdles. A key issue with chatbots is their susceptibility to hallucinations, wherein they generate outputs that appear logical but are factually incorrect or irrelevant to the input context [31,32]. This phenomenon arises from inherent biases, limited real-world comprehension, or constraints in training data. In our investigation, all conversations were initiated independently to prevent contextual interference Furthermore, it is imperative to recognize two significant constraints when considering the utilization of chatbots such as ChatGPT or GEMINI for practical medical applications. Firstly, these platforms are not explicitly tailored or authorized for medical diagnosis, treatment, or decision making [33,34]. While they can furnish general information and responses, they cannot serve as a substitute for professional medical advice or consultation with qualified healthcare providers. Secondly, the efficacy of AI models is contingent upon the quality of input data, necessitating careful manual input of relevant information. Their precision and dependability are greatly influenced by the data they are trained on [35]. Accordingly, our preliminary results also pointed to the possible limitations and the subsequent need for strict observation by trained and experienced medical professionals who should employ a continuous monitorization of performances and biases of LLMs in healthcare, as a basis for their future development [36].

The strengths of our manuscript lie in the pioneering examination of LLMs for maxillofacial trauma triage, offering a cutting-edge perspective in medical technology application and providing valuable comparative insights through real-case scenario analysis. However, it is important to acknowledge limitations, primarily revolving around the exploratory and preliminary nature of the study (e.g., small samples, single-center design, and paucity of quantifying outcomes). Additionally, the study’s reliance on specific LLMs (ChatGPT and GEMINI) may not comprehensively represent the capabilities of other emerging models. In conclusion, we underscore the imperative for future randomized controlled trials (RCTs) and observational studies in clinical settings to rigorously test the hypothesis of the present manuscript. Emphasizing empirical, data-driven approaches, future studies should strive to establish a clear, evidence-based framework for the integration of LLMs in healthcare, ensuring their contributions are both effective and ethically sound in enhancing patient care and medical decision-making processes. Different LLMs may develop unique ‘areas of expertise’ or ‘interpretive biases’ based on their training, ultimately influencing their clinical recommendations. Therefore, a strategic selection of LLMs in clinical settings may be appropriate, where the choice of model could be tailored to the specific nature of the clinical question or the complexity of the case at hand. Moreover, analyzing cases where the models diverge could reveal gaps in their training data or areas where additional context could enhance their performance, as an opportunity for iterative improvement.

## 5. Conclusions

While the evaluation of ChatGPT and GEMINI performance across all clinical scenarios represents a challenge for physicians, this preliminary study illustrates their potential ability to deliver recommendations for maxillofacial trauma cases. Even though moderate agreement with tertiary referral centers and a fair quality of provided information have been retrieved, the limitations identified highlight the necessity for continued supervision and monitoring of these interfaces as they evolve. Future large-scale studies with robust methodologies are warranted to definitively assess the efficacy and safety of integrating LLMs into clinical workflows for maxillofacial trauma management.

## Figures and Tables

**Table 1:** Main characteristics of maxillofacial trauma cases.

	Date of Incident	Age, Sex	Comorbidities	Nature of Injury	Maxillofacial Fractures	Concomitant Injuries
Case 1	October 2023	9, Female	None	Car accident	Orbital floor, nasal, maxillary	None
Case 2	April 2023	15, male	None	Car accident	Mandibular	Cerebral, arm-wrist
Case 3	January 2023	40, male	AIDS	Aggression	Mandibular	None
Case 4	November 2023	51, female	Cerebral Palsy	Domestic Fall	Mandibular	None
Case 5	November 2023	35, male	None	Aggression	Frontal bone, Orbital roof and medial wall, nasal septum	None
Case 6	May 2023	41, male	None	Car Accident	Maxillary, zygomatic, orbit, nasal bones	Cerebral
Case 7	April 2023	28, male	None	Bike accident	Mandibular	Odontological
Case 8	October 2023	64, female	None	Domestic fall	Zygomatic, orbital floor,	Cerebral
Case 9	February 2023	52, male	None	Car Accident	Mandibular	Odontological
Case 10	January 2023	54, male	None	Aggression	Zygomatic, orbital floor,	None

**Table 2:** Triage indication (multidisciplinary evaluation and treatment) of LLMs and tertiary referral center.

	Multidisciplinary Evaluation			Treatment		
	ChatGPT 4.0	GEMINI	Tertiary Referral Center	ChatGPT 4.0	GEMINI	Tertiary Referral Center
Case 1	Ophthalmologist	Ophthalmologist	Opthalmologist	Observation, Orbital floor repair	Orbital floor repair	Orbital floor repair
Case 2	Neurologist, orthopedic	NA	Neurologist, orthopedic	ORIF	ORIF	ORIF, post-operative rehabilitation
Case 3	Infectious diseases	Dentist	NA	ORIF, antibiotics	ORIF; antibiotics	ORIF; antibiotic
Case 4	NA	NA	NA	ORIF	ORIF	ORIF, post-operative rehabilitation
Case 5	Neurologist, ophthalmologist	NA	Neurologist, ophthalmologist	ORIF, antibiotics	ORIF	ORIF, antibiotics, post-operative ICU
Case 6	NA	Ophthalmologist, otolaryngologist	Neurologist, ophthalmologist	Orbital decompression, ORIF	ORIF, soft tissue reconstruction	ORIF, antibiotic
Case 7	Dentist	Otolaryngologist	Dentist	ORIF, teeth splinting	ORIF, post-operative rehabilitation	ORIF, teeth splinting, post-operative rehabilitation
Case 8	NA	NA	Ophthalmologist	ORIF	ORIF	ORIF
Case 9	Dentist	NA	Dentist	ORIF, teeth splinting	Intermaxillary fixation	ORIF, teeth splinting, post-operative rehabilitation
Case 10	NA	NA	Ophthalmologist	ORIF	ORIF	ORIF

Abbreviations: acquired immune deficiency syndrome (AIDS); intermaxillary fixation (IMF); not applicable (NA); open reduction internal fixation (ORIF).

**Table 3:** AIPI and QAMAI Scores results of LLMs.

Metric	GEMINI Mean (SD)	ChatGPT Mean (SD)	*p*-Value
AIPI: Patient Feature	2.83 (0.89)	2.75 (1.27)	0.859
AIPI: Diagnosis	3.30 (0.66)	2.30 (1.32)	0.032 *
AIPI: Additional Examination	1.50 (0.77)	2.00 (1.33)	0.327
AIPI: Treatment	1.86 (0.39)	2.10 (0.57)	0.150
AIPI: Total	9.50 (1.98)	7.60 (2.59)	0.052
QAMAI: Accuracy	3.03 (0.29)	3.10 (0.99)	0.802
QAMAI: Clarity	3.47 (0.36)	3.50 (0.88)	0.902
QAMAI: Relevance	2.90 (0.35)	3.50 (0.88)	0.021 *
QAMAI: Completeness	3.00 (0.47)	2.9 (1.12)	0.802
QAMAI: References	3.07 (0.41)	2.65 (1.11)	0.214
QAMAI: Usefulness	2.93 (0.30)	3.20 (1.01)	0.396
QAMAI: Total	18.40 (1.64)	18.85 (5.37)	0.765

* Significance levels: * *p* < 0.05.

## Data Availability

The data presented in this study are available on request from the corresponding author.

## Supplementary Materials

The following supporting information can be downloaded at: https://www.mdpi.com/article/10.3390/diagnostics14080839/s1.
