# Supplementary material for: The Role of Large Language Models (LLMs) in Providing Triage for Maxillofacial Trauma Cases: A Preliminary Study
Source: Diagnostics (Basel). 2024 Apr 18;14(8):839. doi: 10.3390/diagnostics14080839 (PMC11048758; doi:10.3390/diagnostics14080839)
Supplement: Supplementary file 1 [file diagnostics-14-00839-s001.zip › diagnostics-2964388-supplementary.pdf]

Additional material to *“The Role of Large Language Models (LLMs) in Providing Triage for Maxillo-Facial Trauma Cases: a preliminary study”; by Andrea Frosolini, Lisa Catarzi, Simone Benedetti, Linda Latini, Glauco Chisci, Leonardo Franz, Paolo Gennaro Guido Gabriele; Diagnostic 2024*. The following table shows an example of prompts used and responses generated by LLMs employed in the study.

Prompts	LLM1	LLM2
1) Hi LLM (i.e. Gemini or ChatGPT). You are a maxillofacial surgeon receiving the following patient's documentation. Provide the most appropriate triage process based on the presented information:<br>Medical history: The patient sustained a road accident, he was transported to the emergency department where a maxillofacial examination was carried out which revealed a full-thickness wound of the lingual tip, preternatural mobility of elements 11 and 21, exposure of the mandibular cortex at the level of lower right quadrant. Suture was performed on the lingual wound and the mucosa surrounding element 11.<br>The maxillofacial CT reports: Displaced fracture of the medial and posterior wall of the left maxillary sinus with hemosinus, without involvement of the pterygoid processes or orbital floor. Comminuted fracture with displacement-diastasis of the mandibular body, bilaterally. Specifically:<br>- On the left side, mandibular angle fracture with medial dislocation of the body, involvement of the alveolar process of 3.8, displaced medially; fracture line with both full-thickness transversal and longitudinal courses. The condyle is not damaged.<br>- On the right side, fracture of the body in an intermediate position, also full thickness with prevalent irradiation along the major axis, posteriorly; the alveolar bone is	Triage Process for Maxillofacial Injuries<br>This patient has sustained significant mid-facial and mandibular fractures following a motor vehicle accident. Here's the most appropriate triage process based on the information provided:<br>1. Emergency Management:<br>The initial assessment of airway, breathing, and circulation has likely already occurred in the emergency department. However, due to the recent head injury noted on the follow-up CT scan, a re-evaluation for potential airway compromise is crucial.<br>- Monitor vital signs closely.<br>- Control any ongoing bleeding.<br>- Manage pain effectively.<br>2. Maxillofacial Evaluation:<br>- Assess the occlusion (bite) for any significant misalignment that could further complicate treatment or affect functionality.<br>- Evaluate the stability of the fractured segments in the mandible.<br>- Assess for any sensory deficits in the lower lip or chin due to potential nerve involvement.<br>Examine the exposed mandibular bone for signs of infection.<br>3. Neurosurgical Consultation:<br>The recent head CT scan findings of a small laceration and possible bleeding in the brainstem area necessitate immediate consultation with a neurosurgeon. They will determine the severity of the injury and the need for further management.<br>4. Interdisciplinary Care:<br>Coordinate with the orthopedic team managing the forearm-wrist injury to ensure a comprehensive treatment plan.<br>5. Mandibular Fracture Treatment:<br>Depending on the severity of displacement and the neurosurgical assessment, the next step may involve maxillomandibular fixation (MMF) to stabilize the jaw. This can be achieved with wires, elastics, or interdental screws.<br>Open reduction and internal fixation (ORIF) with plates and screws might be necessary for complex fractures, especially if there's significant displacement or comminution. However, the timing of such surgery may be influenced by the head injury and its management.<br>6. Dental Considerations:	Given the comprehensive medical history and the findings you've shared, this patient presents with complex multi-system trauma necessitating a multidisciplinary approach for optimal care. The triage process for such a patient should be meticulously planned to prioritize life-threatening conditions while simultaneously addressing the various injuries to ensure the best possible outcome. Here's a proposed triage process based on the presented information:<br>### Immediate Post-Accident Phase (Already Completed)<br>1. **Initial Emergency Care**: Immediate life-saving measures, airway management, control of bleeding, and prevention of shock.<br>2. **Primary Assessment and Stabilization**: Conducted upon arrival at the emergency department, focusing on ABCs (Airway, Breathing, Circulation), and addressing any immediate life-threatening issues.<br>3. **Initial Maxillofacial Examination and Management**: Assessment of facial wounds, fractures, and preliminary management (e.g., suture of the lingual wound, stabilization of loose teeth).<br>4. **Imaging and Diagnosis**: CT scans to evaluate the extent of injuries, including the maxillofacial CT and brain CT for neurologic assessment.<br>5. **Admission to Intensive Care Unit (ICU)**: For close monitoring and management of potential complications, including respiratory, circulatory, and neurologic support.<br>### Subsequent Care and Specialist Involvement<br>1. **Multidisciplinary Team Coordination**:<br>- **Maxillofacial Surgery**: Detailed planning for surgical intervention to address the mandibular and maxillary fractures. The fractures described suggest the need for open reduction and internal fixation (ORIF), paying close attention to the dental alveoli and ensuring proper alignment and healing of the jaw.<br>- **Neurosurgery/Neurology Consultation**: Given the evidence of diffuse axonal injury and potential bleeding in the brain, continuous monitoring and evaluation by neurology or neurosurgery specialists are crucial. The interpeduncular cistern bleeding and pontine area focus need close observation for any signs of neurological deterioration.<br>- **Orthopedics**: Follow-up and ongoing care for the forearm-wrist injury, ensuring proper healing and rehabilitation of the limb.<br>- **Otolaryngology (ENT)**: If there are concerns about airway obstruction or complications from the facial injuries.<br>- **Psychology/Psychiatry**: Considering the traumatic nature of the accident, psychological support is crucial for the patient's mental health and recovery.<br>2. **Surgical Planning and Execution**:<br>- Timing of surgeries should consider the patient's overall stability, with life-saving procedures prioritized.<br>- Detailed planning for facial reconstruction, ensuring that functional and aesthetic outcomes are optimized.<br>3. **Rehabilitation and Follow-Up**:

affected in correspondence with the root tips between 4.5 and 4.6. No condylar fractures on this side.<br>The patient was then admitted to the intensive care unit. The day after the trauma, orthopedic colleagues performed conservative treatment for a traumatic injury to the forearm-wrist. 48-hours after the trauma, a follow-up brain CT scan was performed, with a report of "Compared to the previous control of two days ago: a small lacerated-contusive-hemorrhagic focus is noted in the central-pontine area (diffuse axonal damage?). Suspect of minimal bleeding of the interpeduncular cistern. The other previous findings remain unchanged. In particular, the small contusive-hemorrhagic focus in the left basal frontal subcortical area remains unchanged."	Evaluate the preternatural mobility of teeth 11 and 21. Depending on the extent of damage and root involvement, these teeth might require splinting, root canal treatment, or even extraction.<br>A panoramic X-ray or cone-beam CT scan might be necessary for a detailed evaluation of the teeth and their surrounding bone.<br>7. Long-Term Management:<br>Following treatment for the fractures, a comprehensive dental rehabilitation plan might be needed to address any missing teeth or functional limitations.<br>Speech therapy and physical therapy might be necessary for rehabilitation if there's any functional impairment due to jaw movement limitations.<br>Additional Considerations:<br>Due to the head injury, the patient's ability to participate in decision-making might be compromised. Involving a family member or legal representative in discussions might be necessary.<br>Pain management remains crucial throughout the treatment process.<br>Overall, this case requires a multidisciplinary approach involving maxillofacial surgery, neurosurgery, and potentially other specialists depending on the patient's specific condition.	- Early involvement of physical therapy, especially for the forearm-wrist injury, to ensure the best functional recovery.<br>- Regular follow-up appointments for maxillofacial injuries to monitor healing, manage complications, and plan for secondary corrective procedures if necessary.<br>- Continuous neurological assessment to monitor for any progression of brain injuries.<br>4. **Patient and Family Support and Education**:<br>- Providing information about the nature of the injuries, the expected recovery trajectory, and the importance of follow-up care.<br>- Support groups or counseling services to help cope with the psychological impact of the accident and recovery process.<br>Given the complexity of the injuries, a coordinated approach across specialties is essential for managing the patient's care effectively. The priority is to stabilize the patient while planning for the comprehensive treatment of all injuries to minimize long-term sequelae and ensure the best quality of life post-recovery.
2) Please provide references to support your triage choice.	References to Support Triage Choices<br>1. Emergency Management and Maxillofacial Evaluation:<br>These are standard protocols for trauma patients with facial injuries. You can find them in most major maxillofacial surgery textbooks, such as:<br>"Oral and Maxillofacial Surgery" by Fonseca et al. reference book: Rosenthal et al., 2014<br>2. Neurosurgical Consultation:<br>The presence of a head injury with potential brainstem involvement necessitates immediate neurosurgical evaluation. Reference:<br>"Head Trauma" by Narayan et al. reference book: Narayan et al., 2019<br>3. Interdisciplinary Care:<br>Coordinating care with other specialists involved in the patient's treatment is crucial for a holistic approach. This is a general principle of trauma management.<br>4. Mandibular Fracture Treatment:<br>The decision for MMF or ORIF depends on the specific fracture pattern and displacement. Here are some references:<br>"Management of Mandibular Fractures" by Ellis and Christensen reference book: Ellis	The triage and treatment plan for a patient with complex multi-system trauma, including significant maxillofacial injuries, is supported by principles outlined in several key references and guidelines within the fields of trauma surgery, maxillofacial surgery, neurosurgery, and emergency medicine. Below are references that support the triage choices and the multidisciplinary approach to care:<br>1. **Advanced Trauma Life Support (ATLS) Guidelines**:<br>- American College of Surgeons. (2018). *Advanced Trauma Life Support Student Course Manual* (10th ed.). This manual provides a comprehensive overview of trauma assessment and initial management, emphasizing the importance of a systematic approach to the trauma patient, which is critical in the immediate post-accident phase.<br>2. **Maxillofacial Trauma Management**:<br>- Fonseca, R. J., Walker, R. V., Betts, N. J., Barber, H. D., & Powers, M. P. (2013). *Oral and Maxillofacial Trauma* (4th ed.). Elsevier Health Sciences. This text offers in-depth coverage of the management of maxillofacial trauma, detailing surgical approaches and considerations for facial fractures, which supports the outlined steps for surgical planning and execution for maxillofacial injuries.<br>3. **Neurotrauma and Critical Care**:<br>- Bullock, M. R., Chesnut, R., Ghajar, J., Gordon, D., Hartl, R., Newell, D. W., ... & Walters, B. C. (2006). *Guidelines for the management of severe traumatic brain injury*. Journal of Neurotrauma, 23(suppl 1), S1-S106. These guidelines discuss the management of traumatic brain injury, including monitoring and intervention strategies for complications like

	& Christensen, 2017 [invalid URL removed]<br>"Current Therapy in Oral and Maxillofacial Surgery" by Fonseca et al. reference book: Fonseca et al., 2017<br>5. Dental Considerations:<br>Evaluating and managing teeth involved in facial fractures is essential. References:<br>"Oral and Maxillofacial Trauma" by Fonseca et al. reference book: Fonseca et al., 2014<br>"Contemporary Management of Mandibular Fractures" by Peterson reference book: Peterson, 2011<br>6. Long-Term Management:<br>Rehabilitation following maxillofacial trauma might involve various specialists. References:<br>"Maxillofacial Rehabilitation: Principles and Practice" by Robinson et al. reference book: Robinson et al., 2012 [invalid URL removed]<br>Please note: These are just a few references to support the triage choices. Many other resources can provide detailed information on maxillofacial trauma management.	diffuse axonal injury and intracranial hemorrhage, underscoring the necessity for neurosurgical consultation and neurologic assessment.<br>4. **Orthopedic Trauma Management**:<br>- Court-Brown, C. M., Heckman, J. D., McQueen, M. M., Ricci, W. M., & Tornetta, P. (2015). *Rockwood and Green's Fractures in Adults* (8th ed.). Wolters Kluwer Health. This comprehensive reference details the management of fractures, supporting the approach to orthopedic consultation for the forearm-wrist injury.<br>5. **Psychological Support in Trauma**:<br>- O'Donnell, M. L., Creamer, M., & Pattison, P. (2004). Posttraumatic stress disorder and depression following trauma: Understanding comorbidity. *American Journal of Psychiatry*, 161(8), 1390-1396. This study highlights the importance of psychological support for trauma patients, addressing the high incidence of PTSD and depression following traumatic incidents, thus reinforcing the recommendation for psychological or psychiatric support as part of the multidisciplinary care.<br>6. **Interdisciplinary Team Approach in Trauma Care**:<br>- Wisborg, T., Montshiwa, T. R., & Mock, C. (2017). Trauma care systems: A priority for global health. *Bulletin of the World Health Organization*, 95(10), 728. This article emphasizes the importance of a coordinated interdisciplinary team approach in the management of trauma patients, highlighting the positive impact on patient outcomes.<br>These references provide a foundation for the triage and treatment plan described, illustrating the importance of a systematic, multidisciplinary approach to care for patients with complex trauma injuries.
